# Study protocol for the Korean Human Exposure Safety Survey (KoHESS): a national biomonitoring program for food and consumer product safety

**DOI:** 10.4178/epih.e2025060

**Published:** 2025-10-29

**Authors:** Hyeon-Jeong Lim, Sang-Yong Eom, Sun-Haeng Choi, Seonmi Hong, Byung-Sun Choi, Young-Seoub Hong, Kwan Lee, Won-Ju Park, Jae-Seok Song, Nam-Jun Kim, Hyo-Jeong Hwang, Rihwa Choi, Hosub Im, Youn-Seok Kang, Hye-Young Lee, Mi-Ran Kim, Hyunjin Son, Yong-Dae Kim

**Affiliations:** 1 Department of Preventive Medicine, Chungbuk National University College of Medicine, Cheongju, Korea; 2 Chungbuk Environmental Health Center, Chungbuk National University Hospital, Cheongju, Korea; 3 Department of Occupational and Environmental Medicine, Chungbuk National University Hospital, Cheongju, Korea; 4 Department of Preventive Medicine, Chung-Ang University College of Medicine, Seoul, Korea; 5 Department of Preventive Medicine, Dong-A University College of Medicine, Busan, Korea; 6 Department of Preventive Medicine, Dongguk University College of Medicine, Gyeongju, Korea; 7 Department of Occupational and Environmental Medicine, Chonnam National University Hwasun Hospital, Hwasun, Korea; 8 Department of Preventive Medicine, College of Medicine, Catholic Kwandong University, Gangneung, Korea; 9 Department of Food and Nutrition, Sahmyook University, Seoul, Korea; 10 Laboratory Medicine Center, Division of Laboratory Medicine, GC Labs, Yongin, Korea; 11 Institute for Life & Environmental Technology, Smartive Corporation, Hanam, Korea; 12 Environment Division, Eurofins Korea Co., Ltd., Gunpo, Korea; 13 Food Safety Risk Assessment Division, National Institute of Food and Drug Safety Evaluation, Cheongju, Korea; 14 Chungbuk Regional Cardiocerebrovascular Disease Center, Chungbuk National University Hospital, Cheongju, Korea

**Keywords:** Biomonitoring, Hazardous substances, Exposure assessment, Products for human use, Korean Human Exposure Safety Survey

## Abstract

This protocol paper describes the design and methodology of the Korean Human Exposure Safety Survey (KoHESS), a three-year national biomonitoring program (2023-2025). Korea’s Act on Risk Assessment of Products for Human Use necessitates scientific evidence for aggregate exposure assessment and cumulative risk evaluation of hazardous substances in food and consumer products. Existing biomonitoring programs primarily focus on environmental exposures, limiting comprehensive assessment of products regulated by the Ministry of Food and Drug Safety. KoHESS employs repeated cross-sectional surveys targeting approximately 5,000 Koreans aged 3-79 years per cycle, using complex stratified multistage sampling for national representativeness. Data collection includes standardized anthropometric measurements, biological samples (blood, urine), comprehensive exposure source surveys, and 2-day 24-hour dietary recalls. Chemical analyses using liquid chromatography-tandem mass spectrometry and inductively coupled plasma mass spectrometry measure 24 perfluoroalkyl and polyfluoroalkyl substances compounds, 25 phthalate metabolites, and 7 heavy metals, with quality assurance through certified reference materials. KoHESS will provide scientifically robust data for establishing safety standards, enable advanced exposure modeling, facilitate risk assessments reflecting cumulative exposures, support targeted protection for vulnerable populations, and contribute to international biomonitoring networks while promoting preventive hazardous substance management systems.

## INTRODUCTION

The global landscape of environmental health has undergone a fundamental transformation, shifting from fragmented, media-specific approaches to integrated, human-centered evaluations of chemical exposures. This paradigm shift reflects the growing recognition that individuals face simultaneous exposures to multiple chemicals through various pathways, necessitating comprehensive assessment strategies [1,2]. International biomonitoring programs such as the United States National Health and Nutrition Examination Survey (NHANES), the Canadian Health Measures Survey (CHMS), and the European Human Biomonitoring Initiative (HBM4EU) have demonstrated the critical role of population-based exposure data in informing environmental health policy and protecting public health [3,4].

In Korea, the implementation of the “Act on Risk Assessment of Products for Human Use” in January 2022 marks a watershed moment in chemical safety regulation. This legislation mandates comprehensive assessment of aggregate exposure to hazardous substances from food, food contact materials, cosmetics, hygiene products, health functional foods, quasi-drugs, and other products applied to or used by humans [5]. The regulatory framework necessitates robust biomonitoring infrastructure capable of generating scientifically sound exposure data that directly supports risk assessment and management decisions under the Ministry of Food and Drug Safety (MFDS)’s jurisdiction.

The coronavirus disease 2019 (COVID-19) pandemic has profoundly altered lifestyle patterns and consumer behaviors globally, with particularly notable changes in Korea. Studies have documented a 73% increase in food delivery service usage, greater reliance on single-use packaging, altered cooking practices, and intensified use of disinfectants and sanitizers [6,7]. These behavioral shifts have potentially modified exposure patterns to various chemicals, including phthalates from food packaging, perfluoroalkyl and polyfluoroalkyl substances (PFAS) from food contact materials. The health implications of these altered exposure patterns remain largely uncharacterized, necessitating updated biomonitoring approaches that capture contemporary lifestyle patterns.

Existing Korean biomonitoring programs provide valuable data but have limitations. MFDS has conducted ongoing project-based monitoring since 2000 on priority substances [8], including various targeted surveys throughout the 2000s and 2010s, with the Korean Research Project on Integrated Exposure Assessment for Food Safety (KRIEFS, 2010-2014) representing one major initiative focusing on heavy metals and persistent organic pollutants [9]. The Korean National Environmental Health Survey (KoNEHS), an ongoing program initiated in 2009, addresses environmental exposures but excludes MFDS-regulated consumer products [10]. The Korea National Health and Nutrition Examination Survey (KNHANES), established in 1998 as a continuous surveillance system, provides limited biomonitoring within its broader health surveillance framework, primarily measuring selected heavy metals and cotinine [11]. None of these programs comprehensively address emerging contaminants or the full chemical spectrum relevant to food and consumer product regulation.

These limitations create critical regulatory barriers: MFDS cannot establish Korean-specific safety limits for food contact materials, instead relying on foreign reference values that may not reflect local dietary patterns. When elevated exposures occur, agencies cannot identify specific product sources for targeted intervention.

Korean Human Exposure Safety Survey (KoHESS) addresses these problems by integrating biomonitoring of 56 MFDS-regulated chemicals with quantitative source assessment through dietary recalls and product use surveys. This enables establishment of evidence-based safety standards, identification of high-risk products and populations for regulatory prioritization, and transformation from reactive to preventive chemical management. This paper presents the study design and implementation strategies.

## COHORT DESCRIPTION

### Study design

The KoHESS employs a repeated cross-sectional design spanning three years (2023-2025) to assess environmental chemical exposures in the Korean population. For participants under 19 years, parental consent and child assent were obtained according to age-appropriate procedures.

The study design builds upon MFDS’s continuous biomonitoring experience since 2000, including validated questionnaire items and sampling strategies from various project-based programs conducted throughout this period, while expanding scope to address contemporary exposure concerns. This prior experience eliminated the need for extensive pilot testing, allowing resources to focus on implementing enhanced analytical capabilities and quality assurance measures.

KoHESS operates through a multi-institutional framework with six regional universities conducting standardized field surveys. Biospecimens are transported under cold chain management to a central biobank for processing and storage at -75°C with barcode tracking. Quality assurance includes annual standardization training, regular quality control assessments, and individual return of clinical test results to participants (Supplementary Material 1).

### Study population and sampling strategy

The target population encompasses Korean residents aged 3-79 years, stratified by detailed age groups (3-6, 7-12, 13-18, 19-29, 30-39, 40-49, 50-59, 60-69, 70-79 years) to ensure adequate representation across all life stages and capture diverse consumption patterns within adult subgroups. A total of 5,000 participants will be recruited across three phases: Year 1 (2023, n=1,000), Year 2 (2024, n=2,000), and Year 3 (2025, n=2,000).

The sampling strategy employs a complex, stratified, multistage approach designed to ensure national representativeness while capturing regional variations in exposure patterns. In the first stage, 15 metropolitan cities and provinces were selected, excluding Jeju Island and Sejong City due to logistical constraints. The second stage involved selecting 58 cities, counties, and districts using probability proportional to size sampling. In the third stage, 104 township clusters (*eup, myeon, dong*) were extracted, with households serving as the final sampling unit.

Sample size allocation across strata utilized the square root allocation method to ensure adequate representation of all demographic groups while maintaining statistical efficiency. Within each cluster, systematic random sampling with a grid-based approach ensured geographic coverage. For children and adolescents, family-based recruitment was prioritized to enable parent-child exposure comparisons and enhance understanding of household-level exposure patterns (Table 1).

Participants are recruited through professional survey agencies with experience in national health surveys, using various contact methods such as home visits and telephone calls. To encourage participation, all participants receive incentives and their individual clinical test results. Communication strategies emphasize both the national importance of the study and the direct health benefits to participants and their families, while strictly ensuring confidentiality and voluntary participation.

### Ethics statement

The study protocol received approval from the Institutional Review Board of Chungbuk National University (IRB No. CBNU-202302-HRBR-0010). All participants provided written informed consent prior to enrollment.

## MEASUREMENTS

### Data collection procedures

KoHESS is conducted through collaborative research among six universities, each responsible for data collection in their respective regions to ensure comprehensive national coverage. To ensure data consistency across all sites, standardization training is conducted at the beginning of each year for all interviewers. The training covers standardized protocols for anthropometric measurements, questionnaire administration techniques, and quality control procedures.

Data collection is systematically conducted following standardized protocols in the order of anthropometric assessment, biological sample collection, questionnaire, and dietary assessments. First morning urine samples are collected from all participants, while blood samples are obtained only from participants aged 11 years and older. Questionnaires and dietary surveys are completed by participants in advance and reviewed on-site by trained interviewers according to standardized guidelines on the day of the survey. Detailed information for each survey component is presented in Table 2.

#### Anthropometric assessment

Standardized anthropometric measurements provide essential covariates for exposure assessment. Height and weight measurements follow standardized protocols using calibrated equipment. Waist circumference is measured according to NHANES protocol, just above the iliac crest at the end of normal expiration. Blood pressure measurements use automated devices with age-appropriate cuffs after 5 minutes of seated rest.

#### Biological sample collection

The collection of biological samples is designed to balance analytical precision with participant acceptability. Blood specimens are drawn by certified medical technologists or nurses following standardized procedures. Specifically, 12 mL of blood is collected in metal-free serum tubes for PFAS analysis, 6 mL in serum separator tubes for clinical chemistry testing, and 6 mL in metal-free EDTA tubes for heavy metal analysis. Urine samples are primarily collected as first-morning voids; if not feasible, they are collected on-site. Participants are instructed to provide at least 50 mL of urine in pre-washed polypropylene containers. A specimen collection kit, along with detailed instructions and a cold pack, is provided to each participant to maintain sample quality during transport.

The collected biospecimens are used not only for chemical exposure analysis but also for clinical testing to assess participants’ health status. Blood samples are used for complete blood count, serum biochemistry, and liver function tests, providing an overview of general health. Urine samples are analyzed to evaluate renal function and urinary tract health. These clinical test results are individually returned to participants to support personal health management and are also interpreted in conjunction with chemical exposure data to provide a comprehensive basis for health assessment and intervention planning. The specific clinical test parameters are presented in Table 3, along with brief descriptions of their clinical significance.

Biospecimens not subject to immediate analysis are preserved for future research as biobanking resources. These specimens undergo standardized preprocessing and are stored under appropriate long-term conditions. With prior consent, they may be used in related studies. This enables long-term monitoring of chemical exposures and supports research on the development of new biomarkers. The types and quantities of biobanked materials are summarized in Table 4.

To ensure sample integrity, a robust cold chain management system is applied. Real-time temperature monitoring devices are used to continuously track conditions throughout the collection, transport, and storage processes. Samples are transported within a controlled temperature range of 2-8°C using validated refrigerated containers. At local health centers, preprocessing is completed within four hours of collection. Subsequently, samples are transferred to the central biobank, where they are stored at -75°C. The central storage facility is equipped with a dual-refrigeration system and backup power sources to ensure long-term sample stability, even in emergencies.

A barcode-based biospecimen management system is implemented to maintain traceability and quality control. All samples are meticulously tracked from the point of collection through final analysis, and regular quality assessments are conducted to ensure specimen integrity. This systematic biobanking infrastructure is expected to serve as a key national resource for environmental exposure data and to support future epidemiological studies.

#### Questionnaire

The comprehensive questionnaire system builds upon validated instruments from MFDS biomonitoring programs since 2000, updated to address contemporary exposure scenarios. The survey employs a two-component approach: household-level questionnaires capturing shared environmental factors and individual-level questionnaires tailored by age group. Trained interviewers administer questionnaires through face-to-face interviews, with self-administration options for sensitive topics.

The questionnaire encompasses 216-284 items depending on age group, administered through four developmentally appropriate life-stage modules: preschool children (3-6 years), school-age children (7-12 years), adolescents (13-18 years), and adults (≥19 years). The adult module includes age-specific questions to capture varying food and consumer product use patterns across different adult decades, covering (Table 5):

(1) Detailed product use patterns for personal care products (22 types) and household chemicals (14 types), (2) Dietary habits with emphasis on packaged food consumption and cooking methods, (3) Pandemic-related behavioral changes, including disinfectant use frequency and food delivery patterns, (4) Environmental exposures including housing characteristics and occupational factors, (5) Socio-demographic characteristics and household composition, (6) Health status, medical history, and medication use, (7) For women aged ≥13 years: reproductive health history and feminine hygiene product use, and (8) For children: parental exposures (27 items each for maternal and paternal factors) and neurodevelopmental assessments.

While the current phase relies on paper-based questionnaires, a mobile-based survey system is under development for implementation in the second cycle, which will enhance data quality through real-time validation and reduce transcription errors.

#### Dietary assessment

Dietary intake is a key pathway for exposure to environmental pollutants like persistent organic compounds and heavy metals, which bioaccumulate through the food chain. To accurately assess dietary exposure, a validated 24-hour dietary recall method is used, involving participants recalling all foods and beverages consumed in the previous 24 hours [12]. Repeated recalls over different days help improve the accuracy of usual intake estimates and capture individual dietary variation [13-16]. Participants receive instructions and record their intake beforehand, serving as a memory aid for the structured face-to-face interview, which employs a standardized multiple-pass approach to gather detailed information on food types, preparation methods, portion sizes, and eating occasions [12]. Visual aids like food models, household measures, and photographic portion atlases specific to Korean foods enhance portion size accuracy. A second recall is conducted via telephone on a different day following the initial interview to increase data reliability while reducing respondent burden [17]. Quality control is ensured through interviewer training, periodic reliability assessments, and review of a subset of data by senior nutritionists. The collected data are analyzed with CAN Pro 6.0 (http://canpro6.kns.or.kr), which calculates nutrient intakes and links them to contaminant data in foods like seafood, rice, and processed items, enabling comprehensive dietary exposure assessment [18,19].

### Laboratory analysis strategy

The analytical approach prioritizes comprehensive coverage of chemicals relevant to MFDS regulatory mandates while ensuring analytical quality and efficiency. The phased implementation allows for methodological refinement and capacity building:

Year 1 (2023): PFAS analysis for 1,000 participants; Year 2 (2024): PFAS analysis for 2,000 participants, plus retrospective phthalate analysis for all 3,000 participants; Year 3 (2025): PFAS and phthalate analysis for 2,000 participants, plus heavy metals analysis for all 5,000 participants.

The target analytes are presented in Table 6 and were selected based on four criteria: (1) chemicals requiring integrated risk assessment due to widespread exposure through daily consumer products; (2) substances with established human health risks necessitating continuous monitoring for policy development; (3) chemicals of global public concern enabling international comparison; and (4) analytically accessible substances with available methods and infrastructure: 1) 24 PFAS compounds: Legacy compounds (PFOA, PFNA, PFDA, PFUnDA, PFDoDA, PFTrDA, PFTeDA, Br-PFHxS, L-PFHxS, L-PFHpS, Br-PFOS, L-PFOS, PFDS) and emerging alternatives (PFBA, PFPeA, PFHxA, PFHpA, PFBS, various isomers and precursors); 2) 25 phthalate metabolites: Traditional phthalates (metabolites of BBP, DBP, DEHP, DEP, DMP, DnOP, DnHxP, DnPeP) and alternative plasticizers (DINCH, DEHTP, DEHA metabolites); 3) 7 heavy metals: Priority metals (cadmium, lead, mercury) and emerging concerns (chromium, nickel, arsenic, aluminum).

Analytical methods employ liquid chromatography-tandem mass spectrometry with isotope dilution for organic compounds and inductively coupled plasma mass spectrometry for metals. Quality assurance measures include: (1) Analysis of certified reference materials (National Institute of Standards and Technology [NIST] standard reference materials 1957 and 1958 for PFAS, NIST 3673 for phthalates); (2) Participation in international proficiency testing programs (German external quality assessment scheme, laboratory quality management program); (3) Internal quality control samples at 20% frequency; (4) Method detection limits validated for population-level biomonitoring.

## DATA INTEGRATION AND QUALITY MANAGEMENT

### Data processing and harmonization

KoHESS employs a systematic approach to integrate multi-source data comprising questionnaires, dietary recalls, anthropometric measurements, and laboratory results. Data cleaning procedures include: (1) automated range checks with predefined acceptable values for each variable; (2) logical validation of related variables (e.g., pregnancy status consistency with gender/age); (3) identification and coding of implausible values; and (4) double data entry for 10% of questionnaires with discrepancy resolution.

Missing data are handled through standardized protocols: item non-response is coded distinctly from skip patterns, biomarker values below detection limits are flagged separately from true missing values, and patterns of missingness are evaluated to identify potential systematic biases. All data modifications are logged with justification codes to maintain transparency.

### Database structure and variable construction

The integrated database utilizes a relational structure linking five modules (demographic, questionnaire, dietary, clinical, laboratory) through encrypted participant identifiers. Variable naming follows systematic conventions: [Domain][Category][Item]_[Unit] (e.g., DIET_FISH_FREQUENCY_WEEK, LAB_PFOS_SERUM_NGML). Derived variables include cumulative exposure indices and estimated daily intakes calculated by linking dietary consumption with contamination databases.

### Documentation and user resources

To enhance research utility and ensure appropriate data usage, we are developing: (1) Comprehensive Codebook: Variable definitions, valid ranges, missing value codes, and skip patterns with Korean-English descriptions; (2) Statistical Analysis Guidelines: Protocols for handling sampling weights, censored biomarker data, and multiple imputation procedures with SAS/R code examples; and (3) Data Quality Report: Summary of data completeness, detected anomalies, and cleaning decisions for transparency.

These resources will accompany the public dataset, ensuring researchers can appropriately analyze the complex survey data while maintaining comparability with international programs (NHANES, German Environmental Survey [GerES], CHMS) through harmonized variable definitions.

## STRENGTHS AND WEAKNESSES

The KoHESS represents a transformative advancement in Korea’s food and consumer product safety surveillance infrastructure, specifically designed to meet the regulatory requirements of the Act on Risk Assessment of Products for Human Use. By integrating comprehensive biomonitoring with detailed exposure source characterization, KoHESS provides the scientific foundation necessary for implementing aggregate exposure assessment and cumulative risk evaluation mandates under the MFDS’s jurisdiction.

Several key features distinguish KoHESS from existing biomonitoring programs. First, while KoHESS shares the repeated cross-sectional design approach with other programs, its specific focus on MFDS-regulated chemicals fills a critical gap—KoNEHS focuses on environmental pollutants and KNHANES on general health indicators with limited chemical biomonitoring. Second, KoHESS’s comprehensive chemical panel includes 24 PFAS compounds and 25 phthalate metabolites, representing the most extensive assessment of food and consumer product-related contaminants. Third, the integration of biomonitoring with detailed dietary assessment and consumer product use surveys enables direct source attribution, which is essential for MFDS regulatory decisions but not available in other programs. Fourth, the mixed individual and family-based sampling strategy allows both population-level and household-level exposure assessment.

Currently in Korea, KNHANES and KoNEHS provide valuable population health and environmental exposure data [10,11]. Among these, KoNEHS shares the most similarities with KoHESS in terms of biomonitoring design and target chemicals. However, critical differences highlight KoHESS’s unique contributions. While KoNEHS focuses primarily on environmental sources of exposure, KoHESS specifically targets consumer products under MFDS jurisdiction. Most notably, KoHESS incorporates comprehensive dietary assessment through repeated 24-hour recalls with food model-assisted portion size estimation, enabling quantitative linkage between food consumption patterns and chemical body burdens. Additionally, KoHESS’s detailed products for human use surveys—covering 22 personal care products and 14 household chemicals (11 cleaning products and 3 biocides)—provide unprecedented granularity in exposure source characterization. These methodological enhancements allow KoHESS to differentiate between dietary and non-dietary exposure pathways, crucial for regulatory decision-making regarding food contact materials versus other consumer products.

Compared to international programs [3,20,21], KoHESS demonstrates international comparability while featuring a design optimized for Korea’s food and consumer product safety management system. While the United States NHANES and CHMS employ approximately 150-200 questionnaire and 200-250 questionnaire items respectively, focusing on comprehensive health assessments [3,20,22], KoHESS implements a detailed 284-item questionnaire system that enables in-depth evaluation of Korean-specific household chemical product usage patterns and dietary exposure to hazardous substances. This comprehensive questionnaire system helps minimize uncertainty in exposure assessment by systematically collecting product-specific usage frequency and amount data, and provides evidence for identifying high-risk product categories and vulnerable populations. In comparison with the German GerES, KoHESS demonstrates two distinctive approaches. First, while GerES directly measures exposure sources through residential environmental sampling (indoor air, dust, drinking water) [23], KoHESS achieves comparable exposure characterization through comprehensive lifestyle and product use questionnaires while maintaining cost-efficiency. Second, unlike GerES which conducts separate surveys for children and adults [23], KoHESS adopts a household-level integrated assessment approach, designed to analyze correlations between parental exposure factors (27 items each for maternal and paternal factors) and children’s exposures. This family-based approach is utilized to identify familial clustering of exposures resulting from shared use of food and consumer products within households. The comparative characteristics of major domestic and international human biomonitoring programs are presented in Table 7.

KoHESS addresses key analytical limitations in existing biomonitoring programs for public health applications. For example, while current programs can detect elevated phthalate levels in children, they cannot identify specific intervention targets. KoHESS’s integrated dietary assessment and product use surveys enable MFDS to pinpoint high-risk food packaging or personal care products, providing actionable evidence for targeted regulatory measures and consumer guidance rather than general population-level warnings. Additional examples include: (1) Regional heavy metal variations linked to specific local food sources, enabling targeted food safety interventions; (2) Seasonal PFAS patterns identifying contaminated agricultural products; (3) Age-specific exposure profiles guiding age-appropriate safety standards for children’s products.

High-quality biomonitoring data collected through KoHESS will directly support MFDS regulatory activities and public health protection. First, actual Korean exposure levels will inform the establishment of allowable limits for hazardous substances in food and safety standards for substances migrating from food packaging, overcoming previous reliance on foreign data or default assumptions [24]. Second, product-specific usage data facilitates identification of high-risk populations and delivery of targeted safety information. For instance, if certain demographic groups show elevated phthalate exposure linked to specific product categories, MFDS can develop tailored consumer advisories. Third, comprehensive child and parental exposure data support development of stricter standards for children’s products and pregnancy-safe product certifications [25]. Finally, temporal trend monitoring enables rapid response to emerging issues, such as population exposure increases following introduction of new food packaging technologies or chemical substitutions.

From an academic perspective, KoHESS provides rich opportunities for research in food toxicology and chemical risk assessment. The integrated database enables sophisticated exposure modeling studies examining relationships between product use patterns, dietary habits, and biomarker levels. Detailed dietary data support nutritional epidemiology studies examining how food processing and packaging influence both nutrient intake and hazardous substance exposure. The family-based recruitment design enables intergenerational exposure studies critical for understanding developmental origins of health and disease [26]. Particularly, the linkage of repeated 24-hour dietary recalls with biomonitoring data enables quantitative assessment of food-mediated hazardous substance exposure contributions, providing direct evidence for food safety policy development.

The comprehensive exposure data obtained through KoHESS supports a paradigm shift from conventional single-chemical risk assessment to more realistic evaluation approaches incorporating aggregate and cumulative exposures. This transformation is vital for protecting public health in modern societies characterized by complex hazardous substance exposures [27]. The survey enables continuous monitoring of emerging hazardous substances, declining trends in regulated substances, and changes in exposure disparities across population subgroups [28]. Particularly important is the ability to track how Korea’s rapid societal transformations—including dietary westernization, increased consumption of ultra-processed foods, and climate-driven lifestyle changes—influence exposure patterns to hazardous substances.

Looking forward, several enhancements are planned for program sustainability. The mobile-based survey system under development for cycle 2 will improve data quality through real-time validation while reducing transcription errors. Future analytical expansion should consider emerging contaminants such as bisphenols and novel PFAS alternatives [29,30], with the established sample archive enabling retrospective analysis as new chemicals of concern emerge. Although specific international collaboration agreements await formalization, the program design enables future participation in networks such as the Partnership for the Assessment of Risks from Chemicals and World Health Organization Chemical Risk Assessment Network, potentially positioning Korea as a regional leader in exposure assessment. Integration with Korea’s robust health insurance database could enable long-term health outcome tracking, transforming KoHESS into a powerful prospective cohort study.

Despite these strengths, several limitations warrant acknowledgment. The cross-sectional design limits causal inference regarding exposure-health relationships, though the repeated survey structure enables trend assessment. The absence of environmental measurements such as indoor air, dust, and drinking water samples may constrain exposure source verification [31], partially mitigated through detailed questionnaire-based exposure assessment and planned linkage with environmental monitoring databases. Sample size limitations for vulnerable subgroups such as pregnant women or specific occupational groups could be addressed through targeted supplementary studies [32]. Single-point biomarker measurements may not fully capture exposure variability for short-lived chemicals [33], although standardized first morning void collection and detailed dietary recalls partially address this limitation.

Future public release of quality-assured KoHESS data will provide essential baseline information for elucidating complex interactions between consumer product exposures and health outcomes [34]. Integration with international data platforms will enable cross-national comparative studies and meta-analyses, contributing to global hazardous substance safety management frameworks [4]. The comprehensive documentation and analysis guidelines will ensure appropriate data utilization by diverse research communities, fostering innovation in exposure science and regulatory toxicology. Ultimately, KoHESS will function as critical infrastructure supporting the transition from reactive to preventive hazardous substance management systems, with success measured by its contribution to reducing harmful exposures and protecting the health of current and future generations of Koreans.

## DATA ACCESSIBILITY

De-identified KoHESS data will be made publicly available in the future to support research on hazardous substance risk assessment.

Researchers interested in accessing KoHESS data should submit a research proposal to the MFDS or the designated data management center. Access requests will be reviewed by the KoHESS Data Access Committee to ensure compliance with ethical guidelines and data protection regulations. Approved researchers will receive access to: (1) Individual-level de-identified data including questionnaire responses, dietary recall records, anthropometric measurements, and clinical and hazardous substance analysis results; (2) Sample weights and design variables for complex survey analysis; (3) Comprehensive codebooks with Korean-English variable descriptions; (4) Statistical analysis guidelines; and (5) Data quality reports documenting data cleaning procedures.

All data users must agree to terms of use that prohibit attempts to re-identify participants and restrict data usage to research purposes. Information on data access will be made available in the future [website URL forthcoming].

## Figures and Tables

**Table 1.** Distribution of study participants by region, urbanicity, and gender across the three-year study period

Region	Type	Year 1 (2023)	Year 2 (2024)	Year 3 (2025)	Men	Women	Total	%	Recruitment sites
Metro areas									
Seoul	Metro	83	212	305	299	301	600	12.0	12
Busan	Metro	58	192	107	177	180	357	7.1	7
Daegu	Metro	81	-	119	152	152	200	6.1	4
	Rural	-	104	-			104		2
Incheon	Metro	-	150	132	167	166	282	6.7	6
	Rural	51	-	-			51		1
Gwangju	Metro	74	70	93	119	118	237	4.7	6
Daejeon	Metro	81	95	63	120	119	239	4.8	5
Ulsan	Metro	94	-	115	105	104	209	4.2	4
Provinces									
Gyeonggi	Urban	-	349	312	357	352	661	14.2	13
	Rural	48	-	-			48		1
Gangwon	Urban	41	-	64	121	118	105	4.8	2
	Rural	-	98	36			134		3
Chungbuk	Urban	-	-	101	124	122	101	4.9	2
	Rural	50	45	50			145		3
Chungnam	Urban	-	-	104	155	149	104	6.1	2
	Rural	59	88	53			200		4
Jeonbuk	Urban	-	55	-	130	128	55	5.2	1
	Rural	94	109	-			203		4
Jeonnam	Urban	44	-	-	152	148	44	6.0	1
	Rural	45	119	92			256		6
Gyeongbuk	Urban	-	88	47	158	154	135	6.2	3
	Rural	38	44	95			177		4
Gyeongnam	Urban	-	146	-	178	175	146	7.1	3
	Rural	59	36	112			207		5
Total		1,000	2,000	2,000	2,514	2,486	5,000	100	104

Metro, metropolitan.

**Table 2.** Data collection components by age group in the KoHESS, 2023-2025

Components	Contents	Age (yr)			
		≥19	13-18	7-12	3-6^1^
Anthropometric assessment	Height and weight	O	O	O	O
	Waist circumference	O	O	O	O
	Blood pressure and pulse	O	O	O	O
Biological sampling	Blood - PFAS (24 compounds)	O	O	O^2^	-
	Blood - Heavy metals (Cd, Pb, Cr, Ni, Hg, As, Al)	O	O	O^2^	-
	First morning urine - Phthalate metabolites (25 compounds)	O	O	O	O
Questionnaire	Product use - Personal care (22 items)	O	O	O	O
	Product use - Household cleaning products (11 items)	O	O	O	O
	Product use - Biocides (3 items)	O	O	O	O
	Pandemic dietary changes	O	O	O	O
	Functional food/herbal medicine	O	O	O	O
	Environmental exposure	O	O	O	O
	Socio-demographics	O	O	O	O
	Health status - Medical history, medications	O	O	O	O
	Health status - Reproductive history	O	O^3^	-	-
	Health status - Neurodevelopmental assessment	-	O	O	O
Dietary assessment	24-hour recall	O	O	O	O

KoHESS, Korean Human Exposure Safety Survey; PFAS, perfluoroalkyl and polyfluoroalkyl substances; Cd, cadmium; Pb, lead; Cr, chromium; Ni, nickel; Hg, mercury; As, arsenic; Al, aluminum.

1 Parent/guardian completes questionnaire for children aged 3-6 years.

2 Applies from age 11 years.

3 For women aged ≥13 years only.

**Table 3.** Clinical laboratory tests and health screening results provided to participants in the KoHESS

Tests	Test parameters	Test type	Sample type	Clinical significance
Hematological tests	RBC, Hb, Hct, MCV, MCH, MCHC, PLT, WBC, eosinophil	Complete blood count	Whole blood	Blood formation and composition, anemia detection, inflammatory/immune response assessment
Glucose metabolism	HbA1c	HPLC-based assay	Serum	Long-term glycemic control evaluation
Liver chemistries	AST, ALT, γ-GTP	Biochemical assay	Serum	Hepatocellular damage, hepatitis, alcoholic liverdiseaseassessment
Lipid metabolism	Total cholesterol, HDL cholesterol, LDL cholesterol, TG	Biochemical assay	Serum	Cardiovascular disease risk assessment, dyslipidemia evaluation
Uric acid metabolism	Uric acid	Biochemical assay	Serum	Gout and renal function impairment assessment
Thyroid function	TSH, free T4	Biochemical assay	Serum	Thyroid hyper/hypothyroidism evaluation
Immune response	IgE	Immunological test	Serum	Allergic reaction or atopic constitution assessment
Renal function (blood)	Serum creatinine, eGFR	Biochemical assay	Serum	Glomerular filtration capacity and chronic kidney disease risk assessment
Renal function (urine)	Urine creatinine, S.G	Urinalysis	Spot urine	Renal excretory function, hydration status, and urine concentration ability assessment

KoHESS, Korean Human Exposure Safety Survey; RBC, red blood cells; Hb, hemoglobin; Hct, hematocrit; MCV, mean corpuscular volume; MCH, mean corpuscular hemoglobin; MCHC, mean corpuscular hemoglobin concentration; PLT, platelet; WBC, white blood cells; HbA1c, glycated hemoglobin; AST, aspartate aminotransferase; ALT, alanine aminotransferase; γ-GTP, gamma-glutamyl transpeptidase; HDL, high-density lipoprotein; LDL, low-density lipoprotein; TG, triglyceride; TSH, thyroid stimulating hormone; IgE, total immunoglobulin E; eGFR, estimated glomerular filtration rate; S.G, specific gravity; HPLC, high-performance liquid chromatography.

**Table 4.** Biobanking resource production and storage

Resource type	Storage container	Aliquot volume (μL)	Quantity	Storage method
Serum	1.8 mL cryotube	500	>3	Ultra-low temperature freezing (-75°C)
Urine		1,200	6	

**Table 5.** Distribution of questionnaire items by domain and age group in the KoHESS

Domains	Questionnaire items	No. of items by age group (yr)			
		≥19	13-18	7-12	3-6^1^
Products for human use					
Oral hygiene	Toothbrushing frequency, toothpaste amount	3	3	3	3
Medicinal products, cosmetics, biocides	Frequency of use	18	18	18	18
Household chemicals	Frequency of use	11	11	11	11
Hygiene products	Frequency of use, occurrence of skin rash	-	-	-	2
Single-use products	Paper cups, plastic utensils, delivery packaging	14	14	14	14
Cookware	Types and frequency of use	10	10	10	10
Food storage	Container types, microwave use	9	9	9	11
Dietary supplements, herbal medicine	Frequency of intake, duration of use, dose	23	23	23	23
Dietary habits					
Food delivery	Frequency, packaging types	4	4	4	4
Water consumption	Source, amount, container type	7	7	7	7
Cooking methods	Grilling, frying frequency	2	2	2	2
Environmental factor					
Housing	Type, renovation, proximity to roads	10	10	10	12
Indoor environment	Cleaning products, ventilation, type of heating and fuel	11	11	11	11
Outdoor environment	Presence of facilities near the residence	8	8	8	8
Transportation	Mode, fuel type, time of use	2	2	2	2
Socio-demographic					
Household	Size, type, income, parental education, education level, marital status	7	5	5	5
Occupation	Job type, duration, chemical exposure	19	-	-	-
Health behaviors					
Smoking	Status, types of tobacco products, amount, duration, secondhand smoke exposure	10	10	-	-
Alcohol	Frequency, amount	2	2	- -	
Physical activity	Type, frequency, duration	6	6	- -	
Health status					
Medical history	Chronic diseases, diagnosis status, medications	11	11	11	11
Allergies	Use of medications, frequency	2	2	2	2
Women’s reproductive history^2^					
Reproductive health	History of pregnancy, childbirth, breastfeeding, menstruation	20	20	13	-
Feminine hygiene products	Type, amount, usage pattern	7	7	7	-
Maternal parity	Birth weight of the child, weeks, mode	-	-	-	6
Parental exposures^3^					
Maternal	Occupation, smoking, chemical exposure	-	27	27	27
Paternal	Occupation, smoking, chemical exposure	-	27	27	27
Child development^4^	Birth weight, feeding history	-	17	17	-
Neurodevelopment^3^	Korean ADHD rating scale	-	18	18	18
Total items		216	284	259	234

KoHESS, Korean Human Exposure Safety Survey; ADHD, attention-deficit/hyperactivity disorder.

1 Questionnaires completed by parent/guardian.

2 For women aged ≥13 years only.

3 For participants aged 3-18 years.

4 For participants aged 7-15 years.

**Table 6.** Target analytes, analytical methods, and quality assurance measures for the KoHESS

Chemical class	Specific compounds	Matrix	Analytical method	LOD	QA/QC measures
Perfluoroalkyl and polyfluoroalkyl substances (PFAS)					
Legacy PFAS	PFOA, PFNA, PFDA, PFUnDA, PFDoDA, PFTrDA, PFTeDA, Br-PFHxS, L-PFHxS, L-PFHpS, Br-PFOS, L-PFOS, PFDS (n=13)	Serum	LC-MS/MS with isotope dilution	0.01-0.10 ng/mL	NIST SRM (1957,1958), G-EQUAS, matrix spiked QC samples
Emerging PFAS	PFBA, PFPeA, PFHxA, PFHpA, PFBS, 9Cl-PF3ONS, PFOSA, Br-MeFOSAA, L-MeFOSAA, Br-EtFOSAA, L-EtFOSAA (n=11)	Serum	LC-MS/MS with isotope dilution	0.01-0.10 ng/mL	
Heavy metals					
Traditional metals	Cd, Pb, Cr, Ni, Hg, As, Al	Urine	ICP-MS, DMA	0.022-0.598 μg/L	Clinchek 8847, 8848
	Cd, Pb, Ni, Hg	Blood	ICP-MS, DMA	0.018-0.088 μg/L	Clinchek 8840, 8841
Phthalate metabolites					
BBP metabolites	MBzP	Urine	LC-MS/MS	0.056 ng/mL	NIST 3673
DBP metabolites	3OH-MBP, MnBP	Urine	LC-MS/MS	0.057-0.066 ng/mL	NIST 3673
DEHP metabolites	5cx-MEPP, 5OH-MEHP, 5oxo-MEHP, 2cx-MMHP	Urine	LC-MS/MS	0.036-0.095 ng/mL	NIST 3673
DEP metabolites	MEP	Urine	LC-MS/MS	0.068 ng/mL	NIST 3673
DMP metabolites	MMP	Urine	LC-MS/MS	0.032 ng/mL	Matrix spiked QC samples
DnOP metabolites	MCPP	Urine	LC-MS/MS	0.093 ng/mL	NIST 3673
DnHxP metabolites	MnHxP	Urine	LC-MS/MS	0.065 ng/mL	Matrix spiked QC samples
DnPeP metabolites	MnPeP	Urine	LC-MS/MS	0.072 ng/mL	Matrix spiked QC samples
Alternative plasticizers	MCOP, 7oxo-MiNP, MCNP, 2cx-MiDP, MiBP, OH-MiBP, 5oxo-MEHA, 5cx-MEPA, 5cx-MEPTP, 5OH-MEHTP, OH-MiNCH, 1-MEHTM, DEC	Urine	LC-MS/MS	0.023-0.176 ng/mL	NIST 3673, G-EQUAS, matrix spiked QC samples

KoHESS, Korean Human Exposure Safety Survey; PFOA, perfluorooctanoic acid; PFNA, perfluorononanoic acid; PFDA, perfluorodecanoic acid; PFUnDA, perfluoroundecanoic acid; PFDoDA, perfluorododecanoic acid; PFTrDA, perfluorotridecanoic acid; PFTeDA, perfluorotetradecanoic acid; Br-PFHxS, branched perfluorohexane sulfonic acid; L-PFHxS, linear perfluorohexane sulfonic acid; L-PFHpS, linear perfluoroheptane sulfonic acid; Br-PFOS, branched perfluorooctane sulfonic acid; L-PFOS, linear perfluorooctane sulfonic acid; PFDS, perfluorodecane sulfonic acid; PFBA, perfluorobutanoic acid; PFPeA, perfluoropentanoic acid; PFHxA, perfluorohexanoic acid; PFHpA, perfluoroheptanoic acid; PFBS, perfluorobutane sulfonic acid; 9Cl-PF3ONS, 9-chlorohexadecafluoro-3-oxanonane-1-sulfonic acid; PFOSA, perfluorooctane sulfonamide; Br-MeFOSAA, branched N-methylperfluorooctane sulfonamidoacetic acid; L-MeFOSAA, linear N-methylperfluorooctane sulfonamidoacetic acid; Br-EtFOSAA, branched N-ethylperfluorooctane sulfonamidoacetic acid; L-EtFOSAA, linear N-ethylperfluorooctane sulfonamidoacetic acid; Cd, cadmium; Pb, lead; Cr, chromium; Ni, nickel; Hg, mercury; As, arsenic; Al, aluminum; BBP, benzyl butyl phthalate; MBzP, mono-benzyl phthalate; DBP, dibutyl phthalate; 3OH-MBP, mono-3-hydroxybutyl phthalate; MnBP, mono-n-butyl phthalate; DEHP, di(2-ethylhexyl) phthalate; 5cx-MEPP, mono-(5-carboxy-pentyl) phthalate; 5OH-MEHP, mono-(5-hydroxy-2-ethylhexyl) phthalate; 5oxo-MEHP, mono-(5-oxo-2-ethylhexyl) phthalate; 2cx-MMHP, mono-(2-carboxymethylhexyl) phthalate; DEP, diethyl phthalate; MEP, mono-ethyl phthalate; DMP, dimethyl phthalate; MMP, mono-methyl phthalate; DnOP, di-n-octyl phthalate; MCPP, mono-(3-carboxypropyl) phthalate; DnHxP, di-n-hexyl phthalate; MnHxP, mono-n-hexyl phthalate; DnPeP, di-n-pentyl phthalate; MnPeP, mono-n-pentyl phthalate; MCOP, mono-carboxyoctyl phthalate; 7oxo-MiNP, mono-(7-oxo-methyloctyl) phthalate; MCNP, mono-carboxynonyl phthalate; 2cx-MiDP, mono-(2-carboxymethyl-isodecyl) phthalate; MiBP, mono-isobutyl phthalate; OH-MiBP, mono-hydroxyisobutyl phthalate; 5oxo-MEHA, mono-(5-oxo-2-ethylhexyl) adipate; 5cx-MEPA, mono-(5-carboxy-2-ethylpentyl) adipate; 5cx-MEPTP, mono-(5-carboxy-2-ethylpentyl) terephthalate; 5OH-MEHTP, mono-(5-hydroxy-2-ethylhexyl) terephthalate; OH-MiNCH, hydroxy-mono-isononyl cyclohexane-1;2-dicarboxylate; 1-MEHTM, 1-mono-2-ethyl-5-hydroxyhexyl terephthalate metabolite; DEC, diethyl carbonate; DMA, direct mercury analyzer; ICP-MS, inductively coupled plasma mass spectrometry; LC-MS/MS, liquid chromatography-tandem mass spectrometry; LOD, limit of detection; QA/QC, quality assurance/quality control; NIST, National Institute of Standards and Technology; SRM, standard reference material; G-EQUAS, German external quality assessment scheme.

**Table 7.** Comparison of human biomonitoring programs in Korea and other countries

Category	Korea			USA NHANES	Canada CHMS	Germany GerES
	KoHESS	KNHANES	KoNEHS			
Conducting agency	Ministry of Food and Drug Safety	Korea Disease Control and Prevention Agency	Ministry of Environment	Centers for Disease Control and Prevention	Statistics Canada & Health Canada	German Environment Agency
Survey start	2023	1998	2009	1999	2007	1985
Survey cycle (yr)	3	Annual	3	Annual	2	5-10
Current cycle (2025)	1st cycle (2023-2025)	2025 survey	6th cycle (2024-2026)	2025 survey	8th Cycle (2025-2026)	GerES VI (planning)
Sample size	5,000/cycle	-10,000/year	6,000-7,000/cycle	-5,000/year	5,700-6,000/cycle	2,000-5,000
Age range (yr)	3-79	≥1	3-79	All ages	3-79	3-17 (children)
						18-79 (adults)
Sampling method	Stratified multi- stage cluster	Multi-stage stratified cluster	Stratified multi- stage cluster	Complex multistage probability	Multi-stage stratified cluster	Stratified random
Main survey items (2025)	• Questionnaire	• Health interview	• Exposure questionnaire	• Health interview	• Health questionnaire	• Living environment
	• Anthropometric assessment	• Medical examination	• Environmental chemicals in biospecimens	• Physical examination	• Physical measures	• Biospecimen analysis
		• Nutrition survey		• 200+ environmental chemicals	• Fitness tests	• Environmental media
	• Biospecimen analysis	• Environmental chemicals	• Environmental media		• Environmental chemicals	
	• Dietary assessment					
	• Heavy metals (Cd, Pb, Cr, Ni, Hg, As, Al)	• Heavy metals (Pb, Hg, Cd)	• Heavy metals (9 types)	• Heavy metals	• Heavy metals (Pb, Cd, Hg, As)	• Heavy metals
	• Phthalates (25 metabolites)	• Cotinine	• VOCs (benzene, toluene, xylene)	• Pesticides (>40 types)	• Pesticides metabolites	• PAH metabolites
	• PFAS (24 compounds)	• BPA, phthalates	• PAHs metabolites	• PCBs, dioxins	• PCBs	• Phthalates/DINCH
		• Selected POPs	• Pesticides (organophosphates, pyrethroids)	• PFAS (>20 compounds)	• PFAS	• Bisphenols (A, F, S)
				• Phthalates & metabolites	• BPA	• PFAS
			• Phenols (BPA, parabens, triclosan)	• VOCs (>20 compounds)	• Phthalates	• Pesticides
			• Phthalates (16 metabolites)	• PAHs	• Acrylamide	• UV filters
			• PFAS (12 compounds)	• Phenols (BPA, triclosan)	• Environmental tobacco smoke	• Parabens
			• Environmental tobacco smoke	• Flame retardants (PBDEs)	• Plasticizers	• Indoor air pollutants
Key features	• Dietary assessment	• Integrated health-nutrition-environment	• Environmental exposure focused	• World’s largest HBM	• Direct measurement focused	• Europe’s longest HBM
	• Emerging contaminants analysis	• National health statistics	• Bio-environmental simultaneous analysis	• Mobile examination centers	• Fitness testing included	• Special children’s survey

KoHESS, Korean Human Exposure Safety Survey; KNHANES, Korea National Health and Nutrition Examination Survey; KoNEHS, Korean National Environmental Health Survey; NHANES, National Health and Nutrition Examination Survey; CHMS, Canadian Health Measures Survey; GerES, German Environmental Survey; PFAS, perfluoroalkyl and polyfluoroalkyl substances; BPA, bisphenol A; POPs, persistent organic pollutants; VOCs, volatile organic compounds; PAHs, polycyclic aromatic hydrocarbons; PCBs, polychlorinated biphenyls; PBDEs, polybrominated diphenyl ethers; Cd, cadmium; Pb, lead; Cr, chromium; Ni, nickel; Hg, mercury; As, arsenic; Al, aluminum; HBM, human biomonitoring; DINCH, diisononyl cyclohexane-1,2-dicarboxylate; UV, ultraviolet.

## Supplementary materials

Supplementary Material 1. KoHESS operational framework: From field collection to data management
