# Supplementary material for: Study protocol for the Korean Human Exposure Safety Survey (KoHESS): a national biomonitoring program for food and consumer product safety
Source: Epidemiol Health. 2025 Oct 29;47:e2025060. doi: 10.4178/epih.e2025060 (PMC12869138; doi:10.4178/epih.e2025060)
Supplement: Supplementary Material 1. — KoHESS operational framework: From field collection to data management [file epih-47-e2025060-Supplementary-1.pptx]

## Slide 1
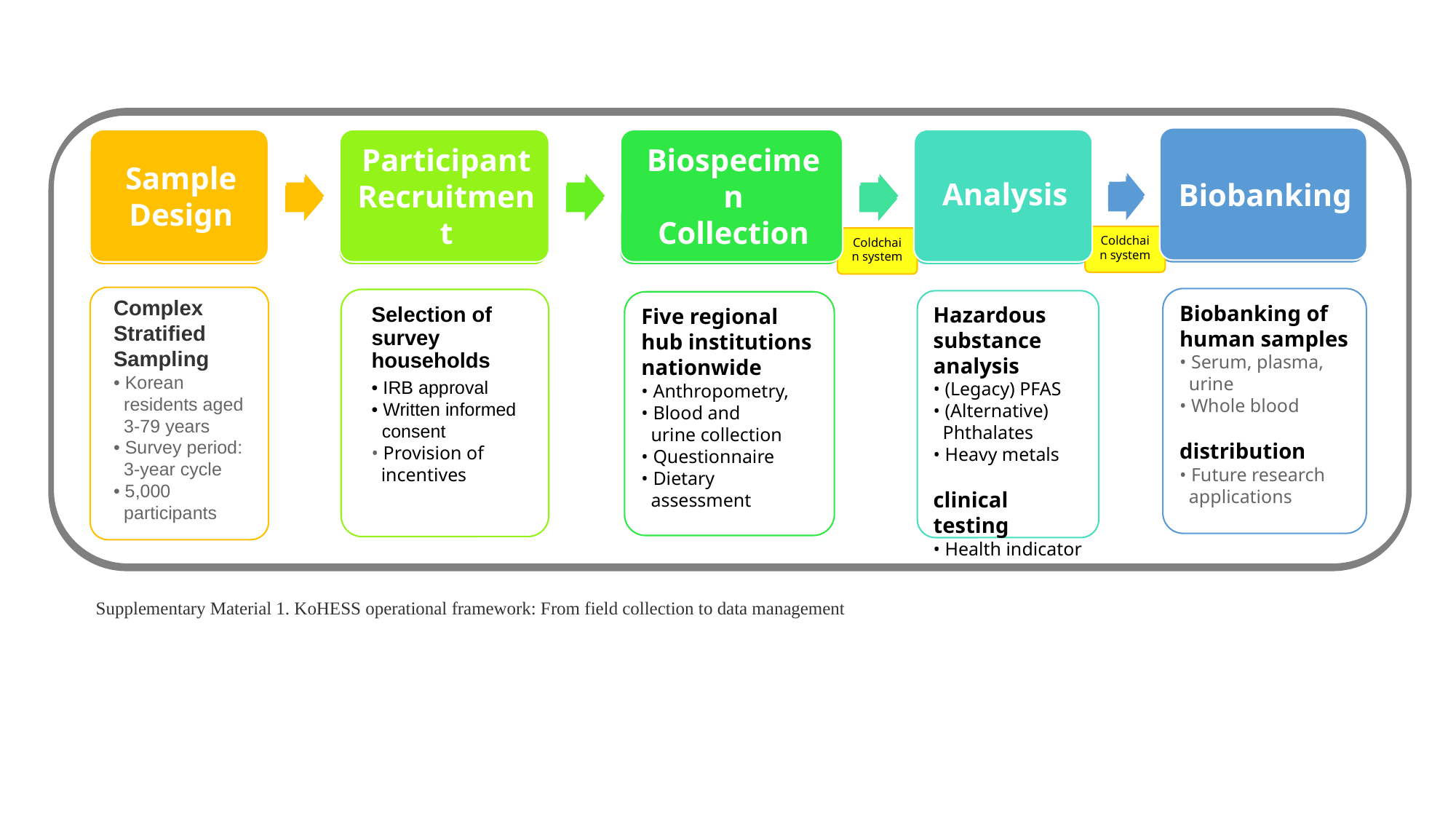

Coldchain system
Coldchain system
Biobanking of human samples • Serum, plasma,
 urine
• Whole blood
distribution
• Future research
 applications
Complex Stratified Sampling
• Korean
 residents aged
 3-79 years
• Survey period:
 3-year cycle
• 5,000
 participants
Selection of survey households
• IRB approval
• Written informed
 consent
• Provision of
 incentives
Hazardous substance analysis
• (Legacy) PFAS
• (Alternative)
 Phthalates
• Heavy metals
clinical testing
• Health indicator
Five regional hub institutions nationwide
• Anthropometry,
• Blood and
 urine collection
• Questionnaire
• Dietary
 assessment
Supplementary Material 1. KoHESS operational framework: From field collection to data management
